# Successful use of the left portal vein as graft for middle hepatic vein reconstruction in left hemihepatectomy: preliminary experience on six cases

**DOI:** 10.1186/s12957-019-1719-0

**Published:** 2019-11-09

**Authors:** Tao Lv, Ling Xiang Kong, Jiayin Yang, Hong Wu, Tianfu Wen, Li Jiang, Jian Yang

**Affiliations:** Department of Liver Surgery, Liver Transplantation Center, West China Hospital, Sichuan University, Chengdu, Sichuan Province China

**Keywords:** Left hemihepatectomy, Portal vein, Hepatic vein, Reconstruction

## Abstract

**Background:**

The purpose of this research was to assess the feasibility of reconstructing the middle hepatic vein (MHV) with resected left portal vein during left hemihepatectomy.

**Methods:**

From January 2014 to January 2018, six patients received left hemihepatectomy combined with MHV reconstruction using the resected left portal vein in West China Hospital. We reviewed the clinical data including patient details, surgical technique, graft patency, and operative results.

**Results:**

All six patients underwent left hemihepatectomy for liver tumors located at left hepatocaval confluence. In these patients, MHV was resected due to tumor invading and reconstructed using the resected left portal vein as graft. The mean operating time was 316 min. Two patients developed complications: one experienced bile leakage and one experienced pleural effusion. No patient developed vascular graft complications. All the grafts remained unobstructed, and no local tumor recurrence occurred during the observation period of 13–41 months.

**Conclusions:**

Our results indicated that the left portal vein was a safe graft for hepatic vein reconstruction. In addition, left hemihepatectomy combined with middle hepatic vein resection and reconstruction using the left portal vein can be performed safely to treat liver tumors located at hepatocaval confluence.

## Background

In recent years, advances in surgical technology have resulted in a great improvement in the survival time of patients who underwent curative liver resection. Due to the invasion of liver tumors, there are a large number of patients that can only be cured with liver resection combined with hepatic vein resection. When the tumor infiltration range is small, a patch reconstruction or primary closure of the hepatic vein is performed. Whereas when the tumor has a wide range of infiltration, hepatic vein reconstruction using a graft is required to prevent vascular stenosis [1]. There have been several researches on using different graft materials for hepatic vein reconstruction, including the internal jugular vein, greater saphenous vein, prosthetic vessels, ovarian vein, and left renal vein [2–6]. However, there are few reports about the use of portal vein grafts isolated from a resected liver. Therefore, the graft patency and results remain uncertain.

In this research, we retrospectively analyzed the clinical data of six patients who underwent left hemihepatectomy for liver tumors located at left hepatocaval confluence. In these patients, MHV was resected due to tumor invasion and reconstructed using the left portal vein.

## Methods

### Patients

From January 2014 to January 2018, 4600 patients underwent various types of hepatectomies at the West China Hospital. Six of these patients underwent left hemihepatectomy with MHV resection and subsequent reconstruction using the left portal vein. The patient characteristics are presented in Table 1. There were two women and four men. The mean age was 55.6 years. In these patients, tumors were located at the junction of the left hepatic vein and the vena cava. The diameters of the liver tumors ranged from 3 to 5 cm. All these patients had hepatocellular carcinomas and were primary surgical cases. According to the Ethics Committee Guidelines at our institution, all the patients signed the informed consent before treatment.

**Table 1:** The clinical features of the patients in this study

Patient number	Sex	Age (year)	Disease	Chile-Pugh score	ICGR 15	Tumor location	Tumor size (cm)	HBV infection
1	F	38	HCC	5	3.6	IVa, II	4	Yes
2	M	56	HCC	5	5.3	IVa	3	Yes
3	M	64	HCC	5	6.4	IVa, II	5	Yes
4	M	70	HCC	6	8.1	IVa, II	4	Yes
5	F	52	HCC	5	4.8	IVa, VIII	4	Yes
6	M	53	HCC	5	4.5	IVa, II	3	Yes

*M* male, *F* female, *HCC* hepatocellular carcinoma, *ICGR 15* indocyanine green retention rate at 15 min

### Preoperative management

**Fig. 1:**
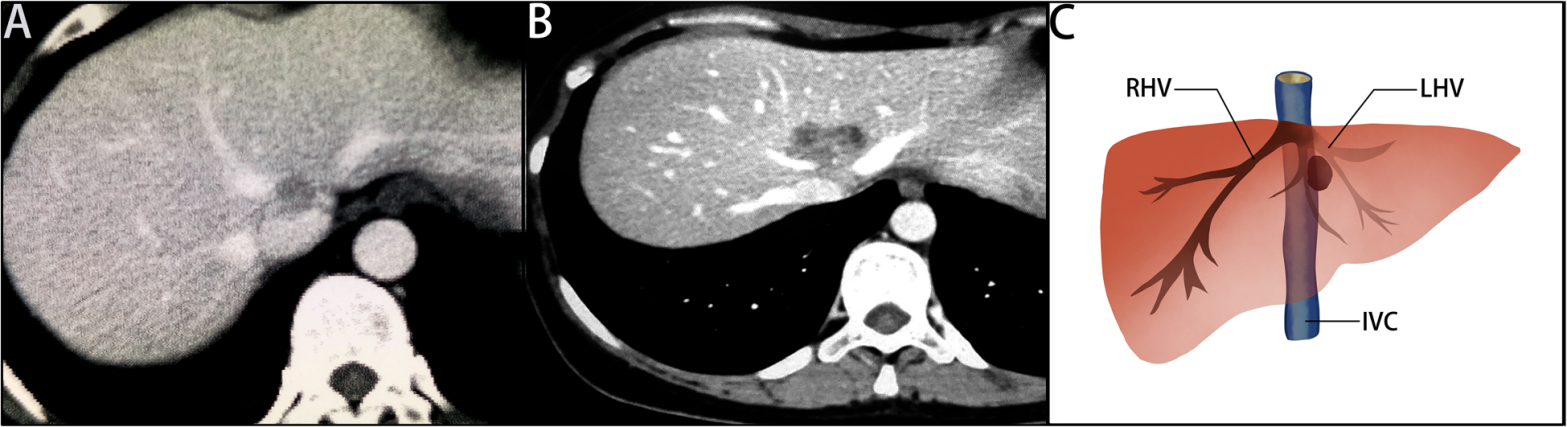
Preoperative CT (**a**, **b**) and diagram (**c**) showing location of the lesions and their relationship with the hepatic veins

Liver function and other routine examinations were performed after admission. Before liver resection, abdominal enhanced computed tomography (CT) scan or magnetic resonance imaging (MRI) was performed to assess the extent of the lesions, gross type, liver volume, and distant metastasis (Fig. 1). Three-dimensional images were reconstructed to evaluate the positional relationships between the blood vessels and tumor. In addition, we used the indocyanine green retention rate at 15 min (ICGR 15) to assess the liver reserve before liver resection. In our center, the indication of left hemihepatectomy was Child-Pugh A, and ICGR 15 < 10%.

### Surgical procedures

**Fig. 2:**
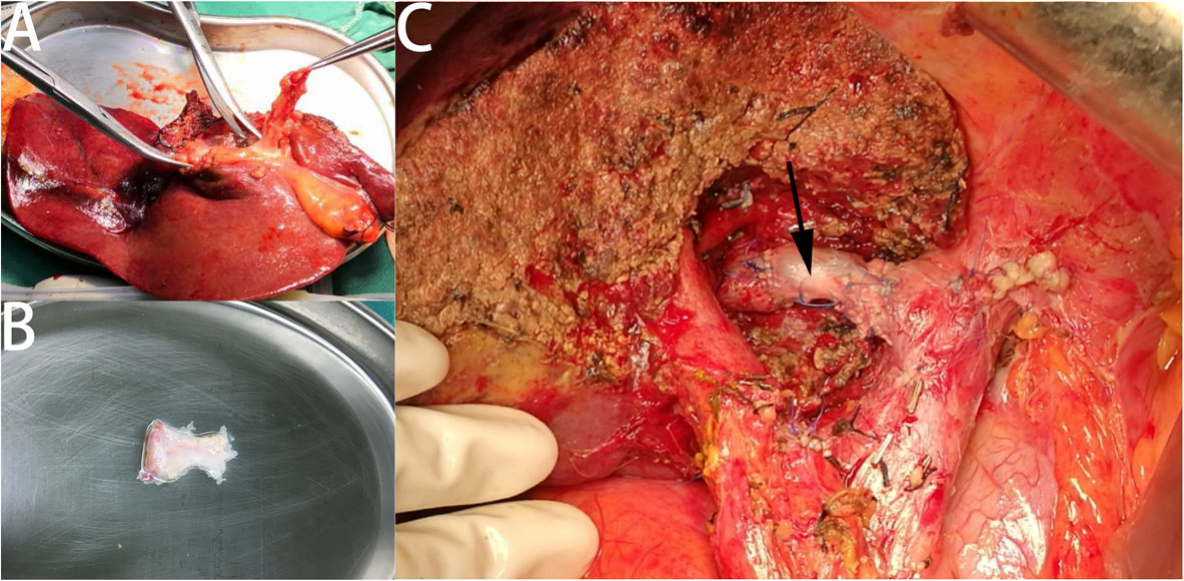
**a**, **b** Isolating and harvesting the left portal vein from the removed liver as a graft. **c** Final appearance of the reconstructed MHV

Our preferred approach was the J-shaped subcostal incision. After freeing the perihepatic ligaments and adequately exposing the left lobe of the liver, we routinely used intraoperative ultrasound (US) to locate the boundaries of the tumor and assess its anatomical relationship with the surrounding important vessels. The liver parenchyma was transected using the Harmonic scalpel (Johnson & Johnson Corp., Princeton, NJ, USA) or CUSA (Valleylab Corp., Somerville, NJ, USA). The intermittent Pringle maneuver was applied to occlude hepatic flow for 20 min, followed by 5 min of liver reperfusion, circularly, until the tumor was completely removed. To obtain sufficient surgical margins, a 2- to 3-cm portion of tumor infiltration of the MHV was resected. During parenchyma transection, the dissected MHV was blocked by vascular clamps. Immediately after resecting the left liver, we isolated and harvested 3 cm of the left portal vein from the dissected liver to use as a graft (Fig. 2). After the hemostasis of liver section, the proximal end of graft was anastomosed continuously to the proximal end of the MHV using 5–0 polypropylene, and then sutured the distal end of graft and the MHV. After the reconstruction, Doppler US was used to confirm the graft patency.

### Postoperative management

**Fig. 3:**
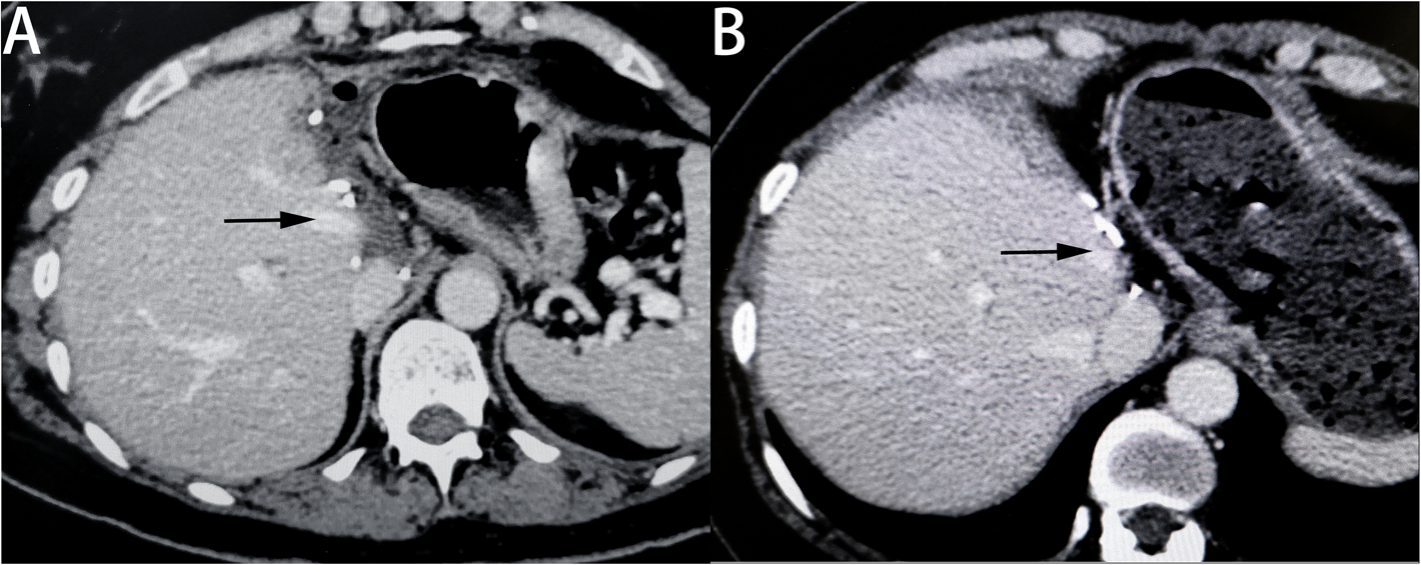
Postoperative enhanced computed tomography. **a** CT showing the patency of the graft at 1 year after the operation (patient 5). **b** CT showing the patency of the graft at 2 years after the operation (patient 2)

Routine postoperative treatments were given after liver resection. From the second day after surgery, all the patients were given low molecular weight heparin sodium (1 mg per kg body weight) for aiming an optimal anticoagulant prophylaxis. After hospital discharge, the patients were treated with warfarin (2.5 mg, qd, po) for 3 months. Ultrasonography or enhanced abdominal CT was performed every month to confirm the patency of the reconstructed MHV in the first 3 months, and post-discharge follow-up studies were conducted at 2-month intervals after that (Fig. 3).

## Results

All the six patients underwent left hemihepatectomy and MHV reconstruction using a left portal vein graft. The clinical outcomes of the patients are presented in Table 2. The mean operating time was 316 min, and mean intraoperative blood loss was 433 ml. Two patients developed complications: one experienced bile leakage and one experienced pleural effusion. The mean postoperative hospital stay was 8.1 days. No patient developed vascular graft complications. Histological invading of the dissected MHV was confirmed in five cases (83.3%). After operation, ultrasonography or enhanced CT was performed to confirm the graft patency and determine recurrence at 1- to 2-month intervals. The mean follow-up was 26.6 months. All the grafts remained patent, and no local tumor recurrence occurred during the observation period of 13–41 months. One patient died of liver failure 31 months after the operation.

**Table 2:** The surgical features and results of the patients in this study

Patient number	Operation time (min)	Blood loss (ml)	Morbidity	POHS (days)	Graft patency (period)	Outcome (period)
1	310	400	None	8	Patent (35 mo)	Alive (41 mo)
2	280	300	None	7	Patent (32 mo)	Alive (37 mo)
3	340	600	Bile leakage	9	Patent (34 mo)	Alive (36 mo)
4	290	400	Pleural effusion	11	Patent (29 mo)	Death (31 mo)
5	350	500	None	6	Patent (18 mo)	Alive (19 mo)
6	330	400	None	8	Patent (12 mo)	Alive (13 mo)

*POHS* postoperative hospital stay, *HIM* histological invasion to the MHV, *mo* months

## Discussion

In our research, we reviewed six cases with reconstruction of MHV using the left portal vein as the graft in left hemihepatectomy. The operations went without any major complication but for only small blood loss. During the follow-up period, all patients were scheduled for an enhanced CT assessment with patency of MHV, and none of the patients had local recurrence. Therefore, left portal vein is a good candidate graft to reconstruct major hepatic veins in selected patients.

Liver resection has become the standard treatment for primary liver malignancies. The critical importance of achieving an R0 resection in liver section is now widely accepted [7, 8]. Due to the surgical approach, left hemihepatectomy is a curative operation for patients with tumors located at the central part of the liver between the left hepatic vein (LHV) and inferior vena cava (IVC) [9, 10]. In case of a tumor invades major hepatic vein, a combined liver and hepatic vein resection is necessary to achieve R0 resection [10, 11].

Opinions differ on the indication for major hepatic vein reconstruction in liver resection. Due to the presence of intrahepatic venous traffic branches, some studies suggest that patients recover from liver congestion after major hepatic vein resection [12, 13]. In contrast, previous studies had found that the reconstruction of hepatic vein was necessary when the congestive volume exceeded 20% of the remnant liver volume (RLV) [14]. In addition, recent studies show that parenchymal abnormalities could result in worse oncological outcomes [15]. Therefore, reconstruction of hepatic veins may be beneficial for patients who had undergone hepatectomy or segmentectomy combined with hepatic vein resection.

Severe hepatic congestion of the right anterior lobe caused by MHV resection during left hemihepatectomy can result in liver dysfunction even when the RLV is adequate. In addition, if the MHV broadly drains the right liver, it remains uncertain whether the congested right liver can increase adequately in volume to compensate for the lack of regeneration [12]. In this case, the reconstruction of major hepatic vein is a crucial step to ensure the safety of patients.

In these six patients, left hemihepatectomy was performed because the hepatic tumors were located at the core part of the liver between the LHV, MHV, and IVC. For the tumors attached to MHV, the resection of MHV was necessary to obtain R0 resection. The left portal vein had enough length and matched the size of major hepatic vein, and the graft was easy to obtain without any additional iatrogenic injury. In addition, preoperative enhanced CT scans revealed the inexistence of tumors invading the portal vein. In consequence, we isolated the left portal vein as a graft to reconstruct the MHV.

Venous reconstruction is now performed skillfully due to the accumulation of experiences from liver transplantation. Recently, various graft materials have been reported for vascular reconstruction in liver section, such as a synthetic artificial graft, cryopreserved vein, and great saphenous vein graft. In our center, we are used to isolate portal vein from the resected liver of recipient as a graft for vascular reconstruction in living donor liver transplantation. Each graft type has its advantages and disadvantages. The artificial blood vessel can be obtained with various sizes and used in various situations. Due to high incidence of infection and obstruction after operation, it is used mainly in reconstruction of large blood vessel. Allograft blood vessel has the same structure and is easy to match the diameter, but it is available only at transplant center because of limited sources. In addition, the homologous nature of these grafts provokes an allogeneic immune reaction, which can result in lower long-term patency rates. Autologous veins are often unavailable because they are limited by size and distance. In addition, harvesting vein grafts leads to vascular congestion, which leads to venous thrombus and dysfunction. Using the left branch of the portal vein as a vascular graft has the following advantages: (1) Comparing with artificial and allograft blood vessel, it does not increase the incidence of infection and rejection. (2) Comparing with autologous veins such as the great saphenous vein, it does not lead to additional trauma or vascular congestion. (3) The left branch of the portal vein is long enough, and its diameter is similar to that of the hepatic vein. (4) The structure of portal vein wall is similar to that of hepatic vein, with high tenacity. The disadvantages are as follows: (1) Removal of the left branch of the portal vein from resected specimens increases the risk of recurrence of liver tumor. (2) The left branch of the portal vein supplies the caudate lobe, so the graft has many branches which need repair. In this study, the operations went without any important complication but for small blood losses (the mean blood loss was 433 ml). The results showed that the technique could effectively reconstruct hepatic vein without increasing blood loss and surgical time. In these six patients, liver tumors locate at the left hepatocaval confluence, which is far from the portal vein. There is no tumor recurrence during the observation period of 13–41 months in all patients. Therefore, this technique should be performed in strictly selected patients. The indications of using the left branch of the portal vein as a graft for hepatic vein reconstruction are as follows: (1) The tumor is located in the second hepatic portal area and invades the middle hepatic vein or left hepatic vein. Therefore, left hepatectomy combined with reconstruction of the middle hepatic vein is needed. (2) The tumor is small in diameter and distant from the first hepatic portal area. (3) No portal vein invasion or portal vein tumor thrombus is found before surgery. This study has shown that the left branch of the portal vein is a perfect graft and this method can be used safely in patients with benign tumor and trauma.

## Conclusions

As far as we know, this study provides the first evidence of left portal vein grafts for major hepatic vein reconstruction in liver resection. Our results suggest that the portal vein is a safe graft and left hemihepatectomy combined with MHV reconstruction using a left portal vein graft can be performed resulting in a good prognosis in selected patients.

## Data Availability

All data are stored in the primary liver cancer clinical database of the West China Hospital. The data in this study are not public but are available from the corresponding author on reasonable request.

## Abbreviations

CT: Contrast computed tomography
ICGR 15: Indocyanine green retention rate at 15 min
IVC: Inferior vena cava
LHV: Left hepatic vein
MHV: Middle hepatic vein
RLV: Remnant liver volume
US: Ultrasound
